# An ultrasound transient elastography system with coded excitation

**DOI:** 10.1186/s12938-017-0375-2

**Published:** 2017-06-28

**Authors:** Xianfen Diao, Jing Zhu, Xiaonian He, Xin Chen, Xinyu Zhang, Siping Chen, Weixiang Liu

**Affiliations:** National-Regional Key Technology Engineering Laboratory for Medical Ultrasound, Guangdong Key Laboratory for Biomedical Measurements and Ultrasound Imaging, Department of Biomedical Engineering, School of Medicine, Shenzhen University, Shenzhen, 518060 China

**Keywords:** Ultrasound transient elastography, Coded excitation, Detection depth

## Abstract

**Background:**

Ultrasound transient elastography technology has found its place in elastography because it is safe and easy to operate. However, it’s application in deep tissue is limited. The aim of this study is to design an ultrasound transient elastography system with coded excitation to obtain greater detection depth.

**Methods:**

The ultrasound transient elastography system requires tissue vibration to be strictly synchronous with ultrasound detection. Therefore, an ultrasound transient elastography system with coded excitation was designed. A central component of this transient elastography system was an arbitrary waveform generator with multi-channel signals output function. This arbitrary waveform generator was used to produce the tissue vibration signal, the ultrasound detection signal and the synchronous triggering signal of the radio frequency data acquisition system. The arbitrary waveform generator can produce different forms of vibration waveform to induce different shear wave propagation in the tissue. Moreover, it can achieve either traditional pulse-echo detection or a phase-modulated or a frequency-modulated coded excitation. A 7-chip Barker code and traditional pulse-echo detection were programmed on the designed ultrasound transient elastography system to detect the shear wave in the phantom excited by the mechanical vibrator. Then an elasticity QA phantom and sixteen in vitro rat livers were used for performance evaluation of the two detection pulses.

**Results:**

The elasticity QA phantom’s results show that our system is effective, and the rat liver results show the detection depth can be increased more than 1 cm. In addition, the SNR (signal-to-noise ratio) is increased by 15 dB using the 7–chip Barker coded excitation.

**Conclusions:**

Applying 7-chip Barker coded excitation technique to the ultrasound transient elastography can increase the detection depth and SNR. Using coded excitation technology to assess the human liver, especially in obese patients, may be a good choice.

## Background

Tissue elasticity and viscosity are closely related to pathological changes. Therefore, quantitative measurement of tissue viscoelasticity has important medical applications. The viscoelasticity measurement technology includes three steps. First, an internal or external dynamic, static, or quasi-static force is applied to the tissue to produce microdeformation or shear waves in the tissue. Second, an ultrasound imaging technique, magnetic resonance imaging (MRI), or optical imaging is used to detect the elastic deformation, the shear wave amplitude, speed, or phase et al. Third, a mathematical model correlating the elasticity, viscosity, density and shear wave information is used to deduce the elasticity and viscosity modulus.

Several ultrasound elasticity techniques with different tissue vibrations have been reported in the past 20 years, including intravascular ultrasound elastography (IVUSE) [1], quasi-static ultrasound elastography [2–4], acoustic radiation force impulse imaging (ARFI) [5, 6], ultrasound vibro-acoustic imaging (USVA) [7, 8], shear wave dispersion ultrasound vibrometry (SDUV) [9], supersonic shear imaging (SSI) [10], external vibration transient elastography [11–13] and so on. Ultrasound-based elastography has the advantages of real-time, noninvasive, low-cost, et al. However, ultrasound-based elastographies have a common defect, in that the detection depth is limited because the ultrasound waves attenuate, and low amplitude shear waves attenuate quickly in the tissue.

One of the clinical applications of ultrasound elasticity technique is to quantitative evaluation of liver, for example, hepatic steatosis, liver fibrosis and cirrhosis. About 50–80% obese patients [14] and 86–96% severely overweight people [15] are liver steatosis. However, ultrasound-based elastographies cannot provide robust shear wave motion detection in the obese patients. The shear wave elastography using the plane wave imaging also suffers from poor penetration [16]. Recently, Echosens develop a probe dedicated to obese patients and controlled attenuation parameter is developed to assess liver steatosis, too [17]. Lai reports that there are about one-third of obese patients have unreliable liver stiffness value measured by vibration-controlled transient elastography by Fibroscan [18]. Thus, it is necessary to develop the ultrasound-based elastography with bigger penetration.

One way to increase the detection depth is to apply coded excitation. There have been several research studies on coded excitation in an elastography technique. Peng and Liu applied coded excitation in the quasi-static elastography and shows that the SNR estimation displacement can be improved, and that the strain noise can be reduced. And they apply chirp-coded pulse detection into a real-time ultrasound elastography system, experimental results also show that the SNR of strain image is increased [19, 20]. Song et al. using a 13-bit Barker code, a short chirp and a long chirp for shear wave detection, results show that the penetration depth can be increased 2, 3, and 4 cm respectively without decreasing the frame rate, field-of-view of plane wave imaging [16]. Although their studies prove that the coded excitation is an important tool to enhance performance of medical ultrasound elastography, little attention has been paid to the effect of using coded excitation on the external vibration transient elastography.

In this study we tested the feasibility of using coded excitation on the external vibration transient elastography. This paper investigates the application of coded excitation on external vibration transient elastography. An external vibration transient elastography system with coded excitation was designed. An elasticity QA phantom (Model 049-CIRS Inc., Norfolk, VA) and in vitro rat livers were used to test the performance of our system. The elasticity QA phantom’s results show that our system is effective, and the rat livers’ results show that penetration depth and SNR are noticeably improved when using Barker coded excitation on the transient elastography system.

## Viscoelasticity mathematical model

For a homogeneous medium, the viscoelasticity mathematical model is as follow [21]:

1 $$c_{s} = \sqrt {\frac{{2\left( {\mu_{1}^{2} + \omega^{2} \mu_{2}^{2} } \right)}}{{\rho \left( {\mu_{1} + \sqrt {\mu_{1}^{2} + \omega^{2} \mu_{2}^{2} } } \right)}}}$$

where *c*_*s*_ is shear wave propagation speed; *μ*_1_ and *μ*_2_ are shear elasticity and shear viscosity of the medium, respectively; *ρ* is the mass density of the medium (most soft tissues *ρ* = 1 kg/m^3^ [22]); *ω* is the frequency of shear wave. However, since not all of the tissues are homogeneous, other models are used to measure elasticity and viscosity, and will result in different elasticity and viscosity values. Suppose shear viscosity is zero, that is, *μ*_2_ = 0, we get the linear elastic model as follows:

2 $$c_{s} = \sqrt {\frac{{\mu_{1} }}{\rho }}$$

where the shear wave propagation speed *c*_*s*_ is called group velocity. If the mass density *ρ* of the tissue is the same, there is one-to-one relation between the group velocity *c*_*s*_ and the shear elasticity *μ*_1_ of the medium. Therefore, in our paper we used shear wave speed on behalf of elasticity.

## Experiment setup

**Fig. 1:**
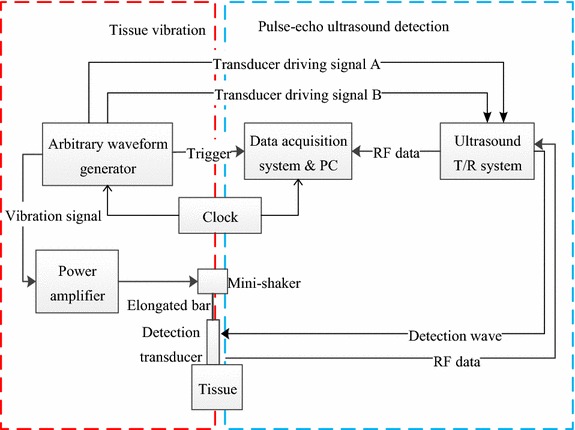
Block diagram of experiment system

The system setup with arbitrary vibration waveform and coded excitation is shown in Fig. 1. The external vibration transient elastography system consists of two parts: tissue vibration and pulse-echo ultrasound detection. The tissue vibration section is used to generate shear waves in medium, and the pulse-echo ultrasound detection section is responsible for detecting weak vibration information. Their details are described as follows:

### Tissue vibration

As shown in Fig. 1 the tissue vibration part consists of an arbitrary waveform generator (LeCroy Arbstudio 1104, Leroy Corp., USA), a power amplifier (Type 2718, B&K, Denmark), a small mechanical vibrator (Mini-shaker Type 4810, B&K, Denmark), a transducer (2 MHz), and an elongated bar. The arbitrary waveform generator can generate a specified vibration signal, and then employ a power amplifier to drive the mechanical vibrator to excite tissue through the elongated bar. The small mechanical vibrator has a frequency range from direct current to 18 kHz, which is enough for elasticity study. The arbitrary waveform generator can produce tissue vibration waveform as required. For example, one period sine wave can be used at different frequencies to excite tissue, similar to suddenly beating the tissue; *N* cycles of sine pulses or a continuous vibration waveform at a frequency can also be used. Different forms of vibration waveform induce different shear wave propagation in the tissue, which is useful for measuring the viscoelasticity of tissue.

### Pulse-echo ultrasound detection

Pulse-echo ultrasound detection shown in Fig. 1 is composed of an arbitrary waveform generator, an ultrasound T/R system, a transducer, a data acquisition system (PCI-9846, ADLINK), and a PC. The arbitrary waveform generator generates two signals to drive the transducer. Likewise, the transducer driving signals produced by the arbitrary waveform generator are very flexible. Consequently, it can achieve either traditional pulse-echo detection or coded excitation. The radio frequency (RF) signals are received by the transducer and captured by the data acquisition system. The sampled RF data are processed offline in the Matlab (The MathWorks, Inc., Natick, MA, USA) environment.

In a conventional Doppler ultrasound system, the transmitting pulse contains several cycles of sine wave whose frequency is identical to the central frequency of the transducer. However, coded excitation in a Doppler ultrasound system must transmit a phase-modulated waveform or a frequency-modulated waveform. Here, the arbitrary waveform generator is chosen to produce a phase-modulated waveform. For the preliminary experiment, a 7-chip Barker code was used for the coded excitation in our study and every chip of the Barker code consisted of four sine waves, abbreviated as 7c4w. The traditional pulse-echo detection emitted four sine cycles with a frequency of 2 MHz. The pulse repetition frequency was 7.4 kHz.

**Fig. 2:**
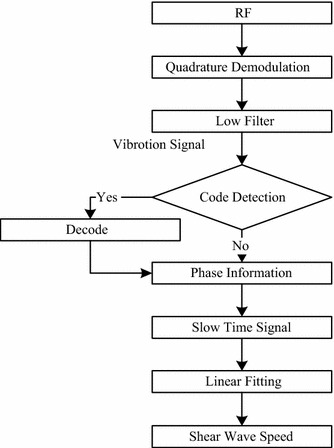
Data processing procedure

Here, the scatter’s vibration with time was described by the slow time signal. The slow time signal’s extracted algorithm was written by referring to the Doppler principle [23]. Shear wave speed can be calculated by the different peak time [21, 24], used in the FibroScan system. The data processing procedure is shown in Fig. 2. When coded excitation mode is used, a matched filter was chosen for decoding, that is, the correlation calculations of the coded sequence and ultrasound echoes through multiplication and summation.

An ultrasound elastography system requires tissue vibration strictly synchronous with pulse-echo ultrasound detection. As shown in Fig. 1, the tissue vibration portion and the pulse-echo ultrasound detection portion work under the same clock source, which was 8 MHz for the whole system. It provided the clock signal to the arbitrary waveform generator and the data acquisition system. Tissue vibration signals, transducer driving signals, and the trigger signal of the data acquisition system were all produced by the arbitrary waveform generator. Therefore, the synchronization of the system could be achieved easily by ensuring that these three signals had a public cycle.

## Evaluation of system performance

### Elasticity QA phantom study

**Fig. 3:**
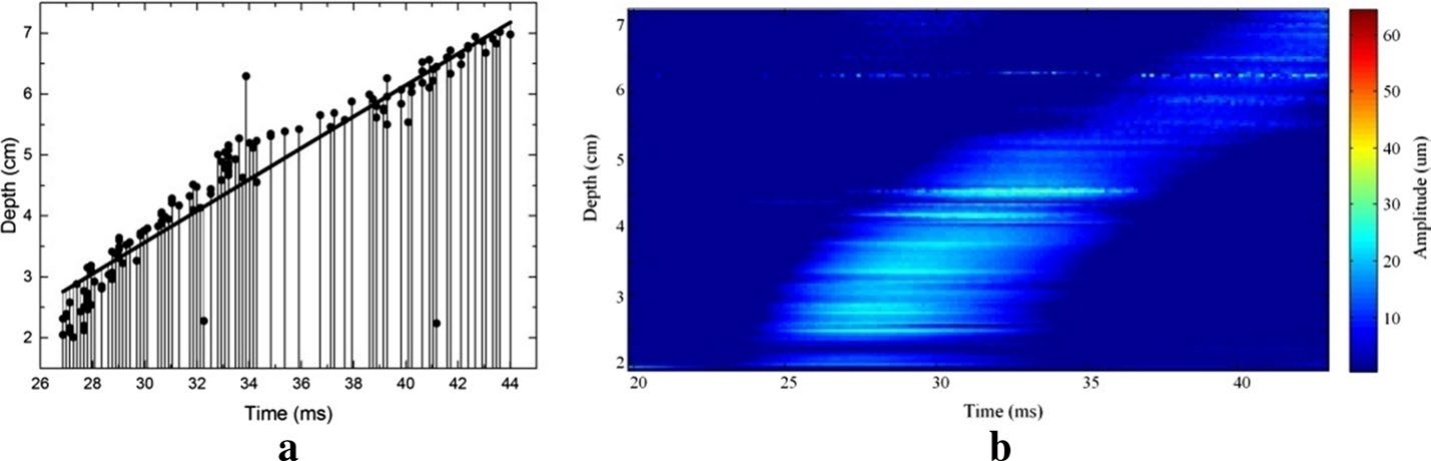
**a** The correspondence between the peak time and depth; **b** The overall trend of the shear wave propagation in the elasticity QA phantom

The developed transient elastography system was used first to measure the elasticity QA phantom, and the results were used to evaluate the system’s accuracy. The tissue vibration signal was a single sine wave, and the pulse width was 8.6 ms; the pulse repetition frequency was 7.4 kHz (results shown in Fig. 3). Figure 3b depicts the overall effect map acquired by drawing the slow time signals of different depths in one picture. This result revealed the vibration propagation process from shallow to deep. Figure 3a depicts the correspondence between the peak time and depth. The deep range was between 2 and 7 cm under the surface. The line was the result obtained by curve fitting, whose slope was 2.7, this meant that the shear wave speed was 2.7 m/s. Under the same conditions we detected ten points, and the shear wave speed result was 2.9 ± 0.18 m/s. The normal value of the elasticity QA phantom is 2.7–3.1 m/s. The elasticity value measured by our system was within the rational range, which indicated that our system was effective.

### Rat liver study

The rat livers were also used to evaluate system performance. Sixteen rats, raised by Guangdong Medical Laboratory Animal Center under identically stable conditions, were used in our experiments. The rat liver was embedded in a phantom, and the distance between the liver and the surface was about 2 cm. The phantom was made from gelatin and water with a 1:9 ratio. All procedures in these studies were approved by Animal Care Committee of Shenzhen University and Guangdong Medical Laboratory Animal Center.

**Fig. 4:**
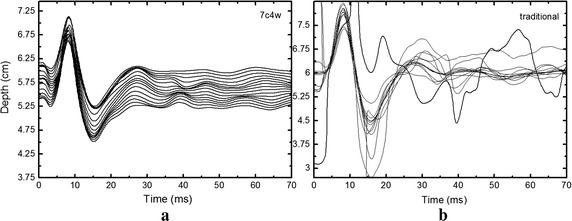
Slow time signals at 5.5–6 cm depth obtained by using different detection methods: **a** is using the 7-chip Barker coded detection, and **b** is using traditional pulse-echo detection

Figure 4 gives the slow time signals obtained by one of the rat liver at 5.5–6 cm depth using 7c4w coded excitation and traditional pulse-echo detection. A single sine impulse with a pulse width of 8.6 ms was used as the vibration signal. Obviously, the slow time signals obtained by using 7c4w coded excitation were more regular. These results show that the 7c4w coded excitation had better stability and anti-interference ability than traditional detection. In fact, coded excitation can increase the signal-to-noise ratio because the white noise in the echoes is reduced through the correlation calculations in the decoding process. Additionally, due to the equipment delay, the main vibration period was about 18 ms, which was very different from the vibration pulse width. The second peak was used to calculate the shear wave speed.

For coded excitation, the corresponding matched filters were used in the offline signals process algorithm. An obvious difference existed relating to the signal amplitude and the initial phase between the results obtained by using 7c4w and traditional detection. The difference has no influence on the measurement of shear wave speed, regardless of which difference (phase or peak time) is used to deduce the shear wave speed, the subtraction operation will remove the bias.

**Fig. 5:**
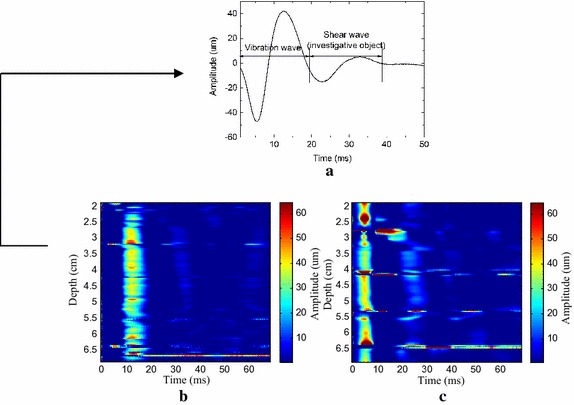
Trend of slow time signals about the rat liver: **a** is the slow time signal at a depth of 3.5 cm; **b** is the overall trend of the shear wave propagation obtained by traditional pulse-echo detection; **c** is obtained using 7c4w code detection

Figure 5 shows the entire trend of slow time signals in one of the rat livers. It was clear that the shear wave propagation was below 5 μm over 4.5 cm in Fig. 5b, while the shear wave propagation process detected by using the 7c4w coded excitation was still about 20 μm in the depth between 4.5 and 5.5 cm in Fig. 4c. The vibration amplitudes detected by 7c4w coded excitation were much bigger than those detected by traditional pulse-echo detection because there were multiplication and summation computation in the decoding algorithm. In addition, as shown by the results in Fig. 5c, the attenuation of the slow time signal amplitude was not obvious when depth was increased, which demonstrated the superiority of the coded excitation in measuring the weak signals.

We detected three points from each rat liver, and then averaged those points after outlier removal. The shear wave speeds of the rat livers in different detection modes were shown in Table 1. A T test showed that the shear wave speed was not significantly different (p > 0.05) between traditional detection and 7c4w detection. Results of sixteen rats show that the 7c4w coded excitation can improve the penetration depth by about 1 cm when the vibration amplitude is only several microns and the SNR is increased about 15 dB.

**Table 1:** Shear wave speed in the rat livers obtained by different detection modes

Detection mode	Shear wave speed (m/s)
Traditional	1.89 ± 0.58
7c4w	2.02 ± 0.98

## Discussion

In our experiments, the tissue vibration signal is a single sine wave, therefore the slow time signal representing tissue vibration is shock attenuation curve. The first peak of the slow time signal is obviously stronger than the second, as shown in Fig. 5a. But the second peak of the slow time signal is used to deduce the shear wave speed. This is because the slow time signal besides the first peak includes the tissue vibration and transducer moving information. If using the first peak of the slow time signal to calculate the shear wave speed, a new algorithm needs to be designed to remove the transducer moving information from the extracted slow time signal. The next work is to design such an algorithm.

This study investigates the feasibility of using coded excitation applied to the external vibration ultrasound transient elastography. The performance of coded excitation and traditional pulse-echo detection are compared using the penetration depth and signal-to-noise ratio (SNR) which are the two main technical specifications of ultrasound elastography. Next, the in vivo study will be done to compare the performance of our ultrasound transient elastography system in quantifying the liver stiffness for the obese. Besides, different coded excitation waveforms or encoding filters need to be investigated to further improve the performance of the coded excitation.

## Conclusions

An ultrasound transient elastography system with coded excitation is developed. The rigorous synchronization of the transient elastography system and coded excitation is executed by using an arbitrary waveform generator. The flexibility of the arbitrary waveform generator ensures that traditional pulse-echo detection, coded excitation, and different tissue vibration waveforms can be realized. Experiment results suggest that higher penetration depth and SNR can be achieved using the 7-chip Barker coded excitation for the ultrasound transient elastography system.

## Abbreviations

SNR: signal-to-noise ratio
MRI: magnetic resonance imaging
IVUSE: intravascular ultrasound elastography
ARFI: acoustic radiation force impulse imaging
USVA: ultrasound vibro-acoustic imaging
SDUV: shear wave dispersion ultrasound vibrometry
SSI: supersonic shear imaging
RF: radio frequency
7c4w: 7-chip Barker code and every chip of the Barker code consisted of four sine waves
